# The Effect of Therapeutic Hypothermia on Ischemic Brain Injury in a Rat Model of Cardiac Arrest: An Assessment Using ^18^F-FDG PET

**DOI:** 10.3390/diagnostics14151674

**Published:** 2024-08-02

**Authors:** Daehee Kim, Woon Jeong Lee, Seon Hee Woo, Hye Won Lee, Bom Sahn Kim, Hai-Jeon Yoon

**Affiliations:** 1 Department of Emergency Medicine, Incheon St. Mary’s Hospital, The Catholic University of Korea, Seoul 06591, Republic of Korea; kim_dae_hee@catholic.ac.kr; 2 Department of Emergency Medicine, College of Medicine, The Catholic University of Korea, Seoul 06591, Republic of Korea; limleeem@catholic.ac.kr (W.J.L.); drme@catholic.ac.kr (S.H.W.); joawony@naver.com (H.W.L.); 3 Department of Nuclear Medicine, College of Medicine, Ewha Womans University, Seoul 07804, Republic of Korea

**Keywords:** positron emission tomography computed tomography, fluorodeoxyglucose F18, cardiac arrest, ischemic brain injury, therapeutic hypothermia

## Abstract

Purpose: Therapeutic hypothermia (TH) is widely acknowledged as one of the interventions for preventing hypoxic ischemic brain injury in comatose patients following cardiac arrest (CA). Despite its recognized efficacy, recent debates have questioned its effectiveness. This preclinical study evaluated the impact of TH on brain glucose metabolism, utilizing fluorine-18-fluorodeoxyglucose (^18^F-FDG) positron emission tomography (PET) in a rat model of CA. Methods: Asphyxia CA was induced in Sprague-Dawley rats using vecuronium. Brain PET images using ^18^F-FDG were obtained from 21 CA rats, who were randomized to receive either TH or no intervention. Of these, 9 rats in the TH group received hypothermia under general anesthesia and mechanical ventilation for eight hours, while the remaining 12 rats in the non-TH group were observed without intervention. We conducted regional and voxel-based analyses of standardized uptake values relative to the pons (SUVR_pons_) to compare the two groups. Results: Survival rates were identical in both the TH and non-TH groups (67%). There was no discernible difference in the SUVR_pons_ across the brain cortical regions between the groups. However, in a subgroup analysis of the rats that did not survive (*n* = 7), those in the TH group (*n* = 3) displayed significantly higher SUVR_pons_ values across most cortical regions compared to those in the non-TH group (*n* = 4), with statistical significance after false-discovery rate correction (*p* < 0.05). Conclusions: The enhancement in SUVR_pons_ due to TH intervention was only observed in the cortical regions of rats with severe encephalopathy that subsequently died. These findings suggest that the beneficial effects of TH on brain glucose metabolism in this asphyxia CA model may be confined to cases of severe ischemic encephalopathy.

## 1. Introduction

Therapeutic hypothermia (TH) is one of the treatment strategies shown to be effective in mitigating ischemic brain injury in comatose patients following cardiac arrest (CA). Early randomized controlled trials conducted in 2002 demonstrated improved outcomes for comatose survivors of out-of-hospital cardiac arrest (OHCA) [1,2]. Subsequently, cardiopulmonary resuscitation guidelines have advocated for the use of TH in comatose survivors of OHCA [3,4]. The primary goal of TH is to attenuate secondary injury associated with reperfusion processes [5,6,7]. This intervention involves a temporary reduction in cerebral metabolic rate, which diminishes the inflammatory response and intracranial pressure, ultimately decreasing the apoptosis of brain cells.

Despite strong guideline recommendations for TH with a target temperature range of 32 °C to 36 °C, recent debates have cast doubt on its efficacy [8]. Critiques focus on the reliance of these guidelines on small, potentially biased trials that include only specific patient subsets. A systematic review encompassing five randomized trials concluded that the evidence supporting TH remains inconclusive, burdened by significant systematic and random errors [9]. Moreover, a more recent systematic review of 17 studies found that TH did not significantly enhance survival or neurological outcomes [10]. Thus, further research into the effects of TH is essential.

In clinical trials, the efficacy of TH is commonly assessed by measuring survival rates, cognitive status, functionality, and quality of life [11]. However, there has been limited exploration of TH’s impact using biomarkers. Only a few studies have indicated the potential utility of blood-based biomarkers in this context [12].

As previously mentioned, TH reduces brain cell loss by mitigating reperfusion injury. Given that brain metabolism primarily relies on aerobic glycolysis, measuring glucose metabolism serves as a direct indicator of brain injury severity. Positron emission tomography (PET) with fluorine 18 fluorodeoxyglucose (^18^F-FDG) is a nuclear imaging technique employed to assess glucose metabolism in vivo. Although FDG PET has been extensively utilized in clinical and preclinical studies for various purposes since 2000, its application in evaluating TH effects remains scarce. While Chevin et al. reported the neuroprotective effects of TH using FDG PET in a hypoxic brain injury model induced by unilateral common carotid artery occlusion, this model differs from the clinical scenario of CA [13]. Nakamura et al. reported differences in FDG uptake based on outcomes in seven comatose patients treated with TH [14]. However, to date, no study has specifically investigated the effect of TH on brain glucose metabolism after CA using FDG PET. In our previous preclinical studies, we demonstrated that assessing brain glucose metabolism with FDG PET provides vital insights into survival and neurological outcomes post-CA [15,16]. In this preclinical study, we evaluated the effect of TH on brain glucose metabolism after CA using FDG PET.

## 2. Methods

### 2.1. Animal Care

Pathogen-free male Sprague-Dawley rats (*n* = 30, weighing 316.32 ± 58.27 g, aged 16 weeks) were acquired from Daehan Bio Link, Eumseong, Republic of Korea. Prior to the experimental onset, the rats underwent a three-week acclimation period under a 12 h light/dark cycle at 50–60% humidity, with unrestricted access to food and water. All experimental procedures adhered to the National Research Council’s 2010 guidelines for the care and use of laboratory animals and received approval from the Institutional Animal Care and Use Committee of Catholic University Medical College (approval number: CIMH2019-006). This study also conformed to the Animal Research: Reporting In Vivo Experiments (ARRIVE) guidelines [17].

### 2.2. Experimental Design

**Figure 1:**
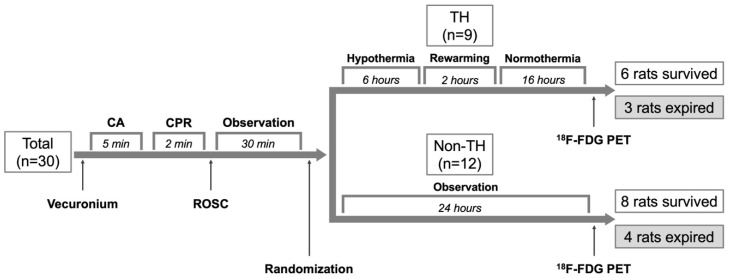
Flow chart of the study design. Abbreviations: CA, cardiac arrest; CPR, cardiopulmonary resuscitation; ROSC, return of spontaneous circulation; TH, therapeutic hypothermia.

The primary objective of this study was to investigate the impact of TH on brain glucose metabolism following CA. The experimental setup is depicted in Figure 1. Briefly, 30 rats underwent asphyxiation to induce CA, with subsequent experiments performed on those achieving return of spontaneous circulation (ROSC). Post-ROSC, rats were randomly assigned to two groups based on whether they received TH: (1) the TH group and (2) the non-TH group. Brain glucose metabolism was evaluated via ^18^F-FDG PET scans conducted 24 h after ROSC in both groups. Survival was monitored daily, and survival duration was documented. Rats that lived for two weeks post-CA were designated as the survived group, while those that died during the follow-up were considered the non-survived group. Morris water maze (MWM) tests were administered to the survived group two weeks post-injury to assess neurocognitive deficits [15]. During the daily survival checks, rats meeting humane endpoint criteria (significant respiratory distress indicated by a respiratory rate below 60 or above 120 breaths per minute, or an oxygen saturation below 90%) were first anesthetized with 4% isoflurane and subsequently euthanized via a gradual CO_2_ filling protocol (30% volume displacement per minute). The surviving rats were anesthetized and euthanized using the same protocol the following day.

### 2.3. Induction of the Cardiac Arrest Rat Model

The induction of the CA rat model involved three key steps: CA, cardiopulmonary resuscitation (CPR), and ROSC. This model was established based on previously described methods with minor modifications [15,16]. Initially, anesthesia was induced with 4% isoflurane and maintained with 1.5% isoflurane. Following tracheal intubation using an 18-gauge catheter, mechanical ventilation commenced using a volume-controlled ventilator (R407; RWD Life Science, Shenzhen, China) with 21% oxygen. Mean arterial blood pressure (MAP) was continuously monitored via a catheter in the femoral artery using an IntelliVue MP40 GCX monitor (Philips, Nashville, TN, USA). To maintain the rectal temperature between 36 and 37 °C, a temperature control system (Homeothermic Monitoring System; Harvard Apparatus, Holliston, MA, USA) was utilized.

After a 5 min observation period, vecuronium (2 mg/kg) was administered intravenously, and both inhalation anesthesia and mechanical ventilation were discontinued [18]. CA was established when MAP dropped below 15 mmHg [16]. Approximately 5 min following CA induction, CPR was initiated with manual compressions at a rate of 200 bpm, accompanied by mechanical ventilation at a fraction of inspired oxygen (FiO_2_) of 1.0. The quality of CPR was evaluated by analyzing the MAP waveforms. At the start of CPR, a single dose of diluted epinephrine (0.04 mg/kg) (Daihan Pharm Co., Ltd., Seoul, Republic of Korea) was administered intravenously. Achievement of a spontaneous MAP exceeding 60 mmHg for at least 30 s was deemed successful ROSC. Animals failing to exhibit ROSC within 2 min of CPR were excluded from subsequent experiments. Following ROSC, mechanical ventilation was continued for an additional 30 min with a FiO_2_ of 0.5.

### 2.4. Therapeutic Hypothermia

Following ROSC, rats were randomized into two groups: the TH group and the non-TH group. Randomization was performed using the random number table method, a straightforward and commonly employed technique in experimental studies that reduces selection bias. Group allocation was determined by whether the TH intervention was administered. The TH protocol utilized in this preclinical investigation was adapted from a previously established method, involving the maintenance of therapeutic hypothermia for 6 h as opposed to the 24 h or longer typically recommended for clinical management post-cardiac arrest [19]. Post-randomization, rats in the non-TH group received mechanical ventilation until spontaneous movement was observed. Following this, tracheal intubation was removed, and the rats were monitored closely for 24 h post-ROSC. Conversely, rats in the TH group were immediately anesthetized with intravenous ketamine–xylazine (ketamine at 1.2 mg/kg/min and xylazine at 0.04 mg/kg/min) following randomization and subjected to mechanical ventilation [20]. Cooling was achieved through surface application of ice bags, with a temperature control system employed to prevent temperatures from falling below the target range. The rats were maintained at a core temperature of 34 ± 0.5 °C for 6 h, gradually rewarmed at a rate of 1 degree per hour for 2 h, and subsequently stabilized at 36 °C for 16 h. Rectal temperature was continuously monitored using the temperature control system throughout the active cooling, maintenance, and rewarming phases. Mechanical ventilation continued for 8 h across these phases. After the rewarming phase, intravenous anesthesia and mechanical ventilation were discontinued, and all monitoring devices were removed 30 min post-rewarming. Following the conclusion of the intervention, survival checks were conducted every 2 h for 16 h without vital signs monitoring, and thereafter at daily intervals for three weeks.

### 2.5. ^18^F-FDG Brain PET Imaging

^18^F-FDG brain PET scans were conducted 24 h post-cardiac arrest induction using a dedicated small animal PET system (microPET-R4; Concorde Microsystems, Knoxville, TN, USA) as previously reported [15,16]. A dose of 37.1 ± 0.8 MBq of ^18^F-FDG was administered intravenously. Subsequent to a 1 h post-injection period, brain PET images were acquired over a 30 min duration. The acquired images were reconstructed with a pixel resolution of 0.2 mm × 0.2 mm and a slice thickness of 0.8 mm using a three-dimensional ordered-subset expectation maximization (3D OSEM) algorithm.

### 2.6. Region-Based and Voxel-Based Image Analysis

An experienced nuclear medicine physician analyzed the brain PET data in accordance with methodologies detailed in prior studies [15,16]. Region-based analysis was facilitated by PMOD 4.2 software (PMOD Technologies, Zurich, Switzerland). Spatial normalization for each brain PET image was achieved using a rat brain PET template and aligned to a volume of interest (VOI) atlas (“Px Rat (W. Schiffer)” provided in PMOD). The mean SUV for the entire cortical VOI, which encompassed the whole cortical areas of specific regions, including the entorhinal cortex, frontal association cortex, insular cortex, auditory cortex, cingulate cortex, medial prefrontal cortex, motor cortex, orbitofrontal cortex, parietal association cortex, retro-splenial cortex, somatosensory cortex, visual cortex, and olfactory cortex, was derived by dividing the radioactivity concentration within the VOI by the administered dose per body weight of the rat. The pons was also included as a reference region. The SUV is indicative of absolute glucose metabolism, albeit susceptible to individual and experimental variations. To mitigate these variations, the SUV for each cortical VOI was normalized against the SUV of the reference region, the pons, to compute the intensity-normalized SUV ratio (SUVR_pons_). The SUVR_pons_ is representative of relative glucose metabolism, facilitating a comparative assessment of metabolic activity across brain regions [18].

Voxel-based analysis utilized SPM12 (Wellcome Trust Centre for Neuroimaging) within MATLAB 2017 (MathWorks, Sherborn, MA, USA). A statistical significance threshold was set at *p* < 0.05, adjusted for the false-discovery rate (FDR), with a minimum cluster size of 50 voxels.

### 2.7. Morris Water Maze Test

An experienced investigator provided preliminary training in the MWM behavioral testing for all subjects. The apparatus consisted of a circular pool with a black interior (1.83 m in diameter, 0.6 m deep), filled with water maintained at 22–24 °C. A transparent Plexiglas escape platform was submerged 1 cm below the water surface, rendering it invisible to the subjects [21]. The pool’s perimeter was decorated with prominent black and white cues for navigation. Both swimming time and distance were monitored and recorded using the Accutrak^®^ computerized tracking system (San Diego, CA, USA). Training consisted of four daily trials over a five-day period. A probe trial was conducted 24 h following the completion of training, and the results were analyzed using analysis of variance (ANOVA). Subjects that demonstrated outlier performance in the probe trial were excluded from further testing. The remaining subjects continued with repeated behavioral assessments two weeks post-injury using the same tracking system.

### 2.8. Statistical Analyses

All statistical analyses were performed using SPSS26.0 (IBM, Armonk, NY, USA). The non-parametric Mann–Whitney U test was utilized to compare SUV differences between the two independent groups (TH vs. non-TH). Survival data were analyzed using Kaplan–Meier estimates, and the survival curves of each group were compared using the log-rank test. Overall survival (OS) was calculated from the date of the induction procedure until death. All results are expressed as medians along with interquartile ranges (IQRs), and *p* values less than 0.05 were considered indicative of statistical significance.

## 3. Results

### 3.1. Course of the CA Rat Model

CA was induced in 30 rats following training in the MWM test. Of these, 21 rats that achieved ROSC were included in the study, representing a 70% resuscitation success rate. Among the 21 rats, 9 received TH treatment, while 12 did not. The body temperature profiles for these rats are presented in Supplemental Figure S1. Survival outcomes were similar between the groups: 6 of the 9 TH-treated rats survived, compared to 8 of the 12 non-TH-treated rats, both groups showing a 67% survival rate. No significant difference in OS was observed between the groups (*p* = 0.848), as detailed in Supplemental Figure S2.

Only survivors were assessed in the MWM test two weeks post-injury. Significant prolongation was noted in the swim time and distance post-intervention [9.3 s (IQR 9.1–9.6) versus 10.1 s (IQR 9.3–10.5), *p* = 0.007; 161.6 cm (IQR 125.8–172.3) versus 186.8 cm (IQR 153.2–225.2), *p* = 0.027]. However, no significant differences were found between the TH and non-TH groups [9.8 s (IQR 9.3–10.2) versus 10.4 s (IQR 9.4–10.9), *p* = 0.475; 185.6 cm (IQR 145.2–217.2) versus 195.8 cm (IQR 155.2–235.2), *p* = 0.755]. Details of the individual MWM results for each group are summarized in Supplemental Table S1.

### 3.2. Distribution of Regional ^18^F-FDG Uptake in Survived and Non-Survived Rats

In non-survived rats, the SUVR_pons_ and SUV for most cortical brain regions were significantly lower than in those that survived, as reported in Table 1 and Table 2. A voxel-based analysis further revealed a global reduction in SUV (FDR-corrected *p* < 0.05, *t* > 2.2978, and cluster extent ≥50 voxels; Supplemental Figure S3) and SUVR_pons_ (FDR-corrected *p* < 0.05, *t* > 2.9343, and cluster extent ≥50 voxels; Supplemental Figure S4) in non-survived compared to survived rats.

### 3.3. Distribution of Regional ^18^F-FDG Uptake According to the Application of TH

#### 3.3.1. Total Group Analysis

**Figure 2:**
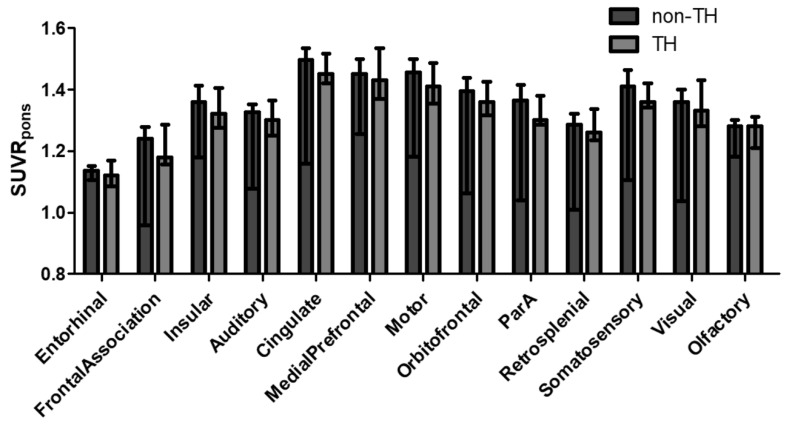
Regional distributions of SUV_pons_ according to the TH intervention in the total group. When the analysis for the total group of 21 cardiac arrest (CA) rats was conducted, the SUVR_pons_ across each brain cortical region showed no differences in regional distribution between the TH (*n* = 9) and non-TH (*n* = 12) groups. Data are presented as medians with interquartile ranges. Abbreviations: SUVR_pons_, standardized uptake value ratio normalized to pons; TH, therapeutic hypothermia.

The analysis encompassed 21 CA rats. The SUVR_pons_ across various cortical regions in the brain, as assessed by PET scans, revealed no differences in regional distribution when comparing the TH group to the non-TH group (Figure 2). Furthermore, voxel-based comparisons confirmed the absence of significant differences in cortical SUVR_pons_ between the groups (uncorrected *p* < 0.001, *t* > 3.5794, and cluster extent ≥50 voxels; Supplemental Figure S5).

#### 3.3.2. Subgroup Analysis: Survived Group

**Figure 3:**
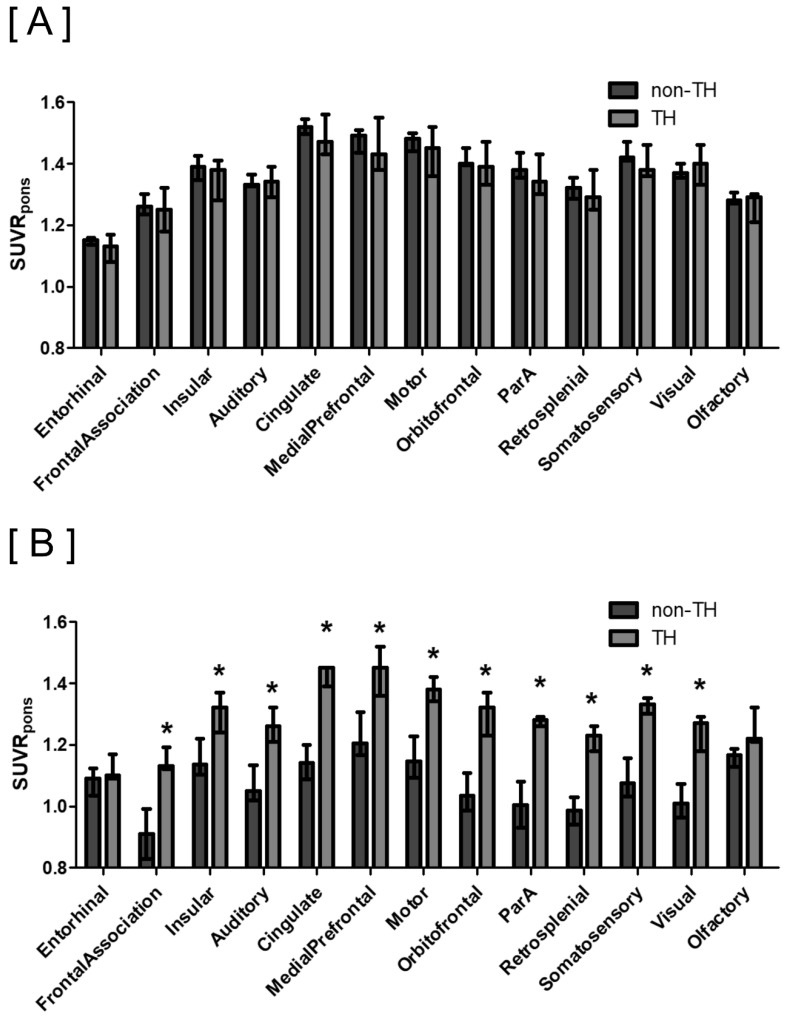
Regional distributions of SUV_pons_ according to the TH intervention in survived (**A**) and non-survived (**B**) subgroups. (**A**) In the subgroup analysis of 14 survived cardiac arrest (CA) rats, the SUVR_pons_ values across each brain cortical region exhibited no differences in regional distribution between the TH (*n* = 6) and non-TH (*n* = 8) groups. Data are presented as medians with interquartile ranges. (**B**) In the subgroup analysis of 7 non-survived CA rats, the TH group (*n* = 3) showed significantly higher SUVR_pons_ than the non-TH group (*n* = 4) in most brain cortical regions (*, *p* < 0.05). Data are presented as medians with interquartile ranges. Abbreviations: SUVR_pons_, standardized uptake value ratio normalized to pons; TH, therapeutic hypothermia.

This subgroup analysis included 14 surviving CA rats. Similarly, the SUVR_pons_ of cortical regions on PET scans indicated no significant regional distribution differences due to the TH intervention in the survived group (Figure 3A). Voxel-based comparisons also showed no significant differences in cortical SUVR_pons_ between the TH group and the non-TH group (uncorrected *p* < 0.001, *t* > 3.9296, and cluster extent ≥50 voxels; Supplemental Figure S6).

#### 3.3.3. Subgroup Analysis: Non-Survived Group

**Figure 4:**
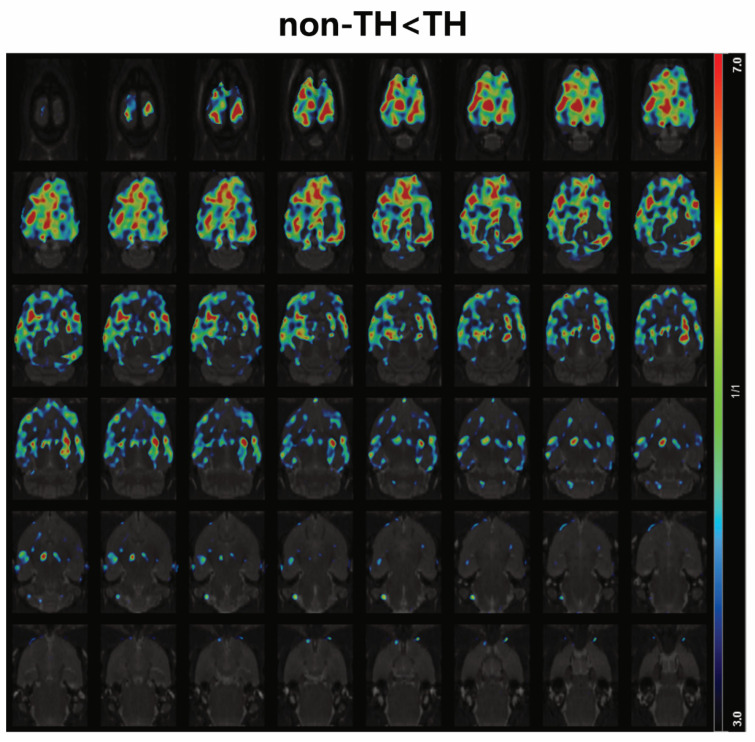
The statistical parametric map of “non-TH < TH” in a non-survived subgroup. The statistical parametric map comparing “non-TH < TH” derived from ^18^F-FDG PET scans normalized to the pons revealed a global increase in relative glucose metabolism in TH rats within the non-survived subgroup (FDR-corrected *p* < 0.05, *t* > 3.3739).

The analysis focused on seven non-survived CA rats. Contrary to other groups, PET scans revealed that most cortical regions displayed varied regional distributions as a result of TH intervention (Figure 3B). Specifically, in the frontal, insular, auditory, cingulate, medial prefrontal, motor, orbitofrontal, parietal, retro-splenial, somatosensory, and visual cortices, three rats in the TH group exhibited significantly higher SUVRs compared to four rats in the non-TH group (all *p* < 0.05). Voxel-based comparisons further demonstrated a global increase in SUVR_pons_ across most cortical regions in the TH group relative to the non-TH group, with statistically significant differences (FDR-corrected *p* < 0.05, *t* > 3.3739, and cluster extent ≥50 voxels; Figure 4).

## 4. Discussion

In this preclinical study, we explored the effects of TH on brain glucose metabolism following CA. The rate of ROSC achieved was 70%, aligning with figures reported in earlier research [18]. Both the TH and non-TH groups displayed identical survival rates at 67%. Furthermore, no differences were observed in either absolute SUV or relative SUVR_pons_ in terms of regional distribution attributable to the TH intervention. However, in a subgroup analysis of the seven non-surviving CA rats, a significant elevation in SUVR_pons_ across the neocortex was noted in the TH group compared to the non-TH group.

Previous studies have indicated selective brain vulnerability to global ischemic insult following CA, with the cortex exhibiting high susceptibility and subcortical deep structures, such as the brainstem, demonstrating less vulnerability [22]. This regional disparity in susceptibility can lead to a pronounced reduction in metabolic consumption in the neocortex relative to the whole brain or subcortical deep structures, potentially indicating a severe degree of encephalopathy [23,24]. In our study, we utilized FDG PET to measure relative glucose metabolism by dividing the regional absolute SUV by the SUV of a reference region—in this case, the pons of the brainstem. Previous findings suggest that neocortical glucose metabolism, adjusted for the SUV of the pons (SUVR_pons_) or whole brain (SUVR_WB_), effectively predicts the severity and prognosis of brain injury post-CA [13,14]. Additionally, the use of SUVR_pons_ as an indicator allowed us to minimize the influence of variables such as body temperature on our results, thereby enhancing the reliability of our findings.

Several preclinical studies have investigated post-CA brain metabolism using ^18^F-FDG PET. However, these studies vary in the timing of FDG PET imaging and the analytical indices employed. Li et al. observed a reduction in cerebral glucose metabolism, quantified by SUV corrected for blood glucose level, at 4, 24, and 48 h post-ROSC [25]. Conversely, Zhang et al., conducting PET imaging at 72 h post-ROSC, noted increases in relative metabolism, assessed by regional ^18^F-FDG uptake normalized to the liver, in post-CA mice compared to control animals [26]. Additionally, Putzu et al. detected a decrease in relative glucose metabolism in the neocortex alongside an increase in posterior regions such as the midbrain, pons, and cerebellum, with regional ^18^F-FDG uptake normalized to the whole brain, during PET scans conducted 50 min post-ROSC [19]. Our previous work, informed by existing research in this field, conducted FDG PET at 3 h post-ROSC and identified variations in relative glucose metabolism correlating with neurological outcomes. Interest has grown in utilizing PET imaging to evaluate treatments during the ROSC phase following CA. Nonetheless, a gap remains due to the lack of preclinical or clinical studies exploring this potential application. While Chevin et al. documented the neuroprotective effects of TH using FDG PET in a hypoxic brain injury model induced by unilateral common carotid artery occlusion—a model distinct from our clinical scenario of CA and ROSC [13]—the study by Nakamura et al. more closely resembles our clinical context. They found that among seven comatose patients treated with TH, the three with poor outcomes exhibited reduced FDG uptake compared to the four with favorable outcomes [14]. However, their findings emphasized outcome differences post-TH treatment, contrasting with our study’s design, which compares PET findings between non-TH and TH groups to elucidate TH’s effects. Moreover, our study not only provides quantitative analyses across different brain regions but also incorporates voxel-based SPM analyses, offering a comprehensive evaluation of the disparities in brain glucose metabolism. Therefore, this research is the first preclinical study to assess the impact of TH post-cardiac arrest using ^18^F-FDG PET imaging.

Building on prior studies of post-CA brain metabolism using ^18^F-FDG PET, it is crucial to determine the appropriate timing of PET imaging to align with the research objectives. This is due to the dynamic nature of cerebral glucose metabolism changes occurring post-CA. The goal of this study was to assess the immediate effects of TH on cerebral glucose metabolism; therefore, FDG PET was conducted immediately following the TH intervention.

Our findings indicate a significant decrease in relative SUVR_pons_ in the non-surviving rats compared to the survivors (Supplemental Figure S4). Notably, improvements in relative glucose metabolism post-TH were exclusively observed in the non-surviving subgroup (Figure 3B). In the survived group, which did not require additional interventions, such as ventilator support, and displayed neurological impairments in the 2-week post-injury MWM test following ischemic injury from reduced brain perfusion, mild hypoxic ischemic encephalopathy was presumed. In these cases, TH did not influence glucose metabolism. Conversely, in the non-survived group—presumed to have experienced severe hypoxic ischemic encephalopathy, defined by their non-survival during the monitoring period post-ischemia—TH was observed to improve neocortical glucose metabolism. However, these effects of TH did not translate into improved survival outcomes, as all subjects died irrespective of receiving TH. This highlights that while TH may enhance cerebral metabolic parameters in cases of severe hypoxic ischemic encephalopathy, it does not necessarily improve survival. These observations, particularly from the non-survived subgroup, must be approached with caution due to potential issues with reproducibility and generalizability arising from the limited sample size. Further research involving larger cohorts is essential to substantiate and extend these preliminary findings.

Our findings contribute to the growing body of preclinical evidence challenging the efficacy of TH following CA. Dankiewicz et al. conducted a randomized clinical trial comparing the effects of targeted hypothermia and normothermia, revealing no significant differences in neurological or survival outcomes between the two groups [11]. Similarly, Garrido et al. conducted a systematic review of 17 randomized clinical trials conducted from 2016 to 2020, aiming to evaluate the effectiveness of TH in patients post-CA [10]. Their analysis also concluded that TH does not enhance neurological or survival outcomes in either adult or pediatric patients. Despite these findings, current international guidelines continue to endorse the immediate initiation of TH for all unconscious patients post-CA, irrespective of the severity of their encephalopathy, to optimize functional and neurological recovery [8].

In contrast to the results in non-surviving rats, no differences were observed in the relative SUVR_pons_ following TH application in surviving rats. This suggests that TH does not affect the improvement of relative glucose metabolism in mild hypoxic ischemic encephalopathy. Furthermore, neurocognitive assessments conducted two weeks after CA showed no differences between the TH-treated and non-treated groups, indicating that TH does not influence long-term neurological and survival outcomes. These findings corroborate our prior research on the prognostic value of neocortical relative glucose metabolism following CA.

To summarize, TH may not be effective for mild encephalopathy following CA, as there were no significant differences in brain glucose metabolism with or without TH in the CA rats that survived. Conversely, in cases of severe encephalopathy, TH was observed to increase neocortical relative metabolism. However, the efficacy of TH in these instances was limited, as it did not substantially improve unfavorable outcomes. It is crucial to highlight that these findings are specific to the groups of animals with either mild or severe encephalopathy, while the effects on those with moderate encephalopathy remain undetermined. Therefore, further research is necessary.

One limitation of this study is the absence of a comprehensive assessment of the injury spectrum following ischemic insult, due to the lack of ex vivo brain tissue analysis. Animals classified with severe injuries that resulted in mortality were found deceased after some time had passed. This delay made perfusion, which must be performed immediately post-sacrifice, unfeasible, and, consequently, tissue processing was not possible. The high mortality rate and severe injury in the selected animal model likely contributed to variability, even among the survived group with minor injuries. However, no distinct infarctions or other abnormalities were noted in brain tissues obtained from surviving animals classified with mild injury at the conclusion of the 2-week MWM test, suggesting a minimal likelihood of bias in the results for this group.

Second, the absence of a moderate encephalopathy group represents a limitation of this study, which may be attributed to variations in the methods used for CA induction. CA etiology is categorized into asphyxia or non-asphyxia types. In asphyxia-related CA, abrupt oxygen depletion occurs due to direct obstruction of the airway and/or neck vessels, leading to a higher incidence of hypoxic brain injury and increased mortality compared to non-asphyxia CA, where cardiac arrest precedes oxygen depletion [27]. In this study, we employed vecuronium to induce CA, mimicking asphyxia CA, as it causes respiratory muscle paralysis. Inducing non-asphyxia CA through electrical initiation of ventricular fibrillation might result in less severe injuries compared to the observed outcomes and warrants further investigation. It is important to acknowledge that our findings are specific to the asphyxia CA model and should be interpreted and applied with caution. Future studies should evaluate the effectiveness of therapeutic hypothermia across different CA models.

Third, the SUV may not accurately reflect the true glucose utilization rate, as it can be influenced by blood glucose levels and other independent factors [28]. Precise quantification of brain glucose utilization in FDG PET can be achieved through the cerebral metabolic rate of glucose (CMR_glc_), using a dynamic quantitative approach. However, this method is technically demanding and time-consuming, requiring arterial blood sampling at multiple time points and complex mathematical modeling. Nevertheless, several studies have shown that SUV, despite being non-invasive, simple, and robust, correlates well with the CMR_glc_ rate [29,30].

Fourth, anesthesia conditions were designed to differ between the TH and non-TH groups to more closely mimic current clinical practice in the treatment of cardiac arrest patients. During the 8 h hypothermia period, the TH group received anesthesia, whereas the non-TH group was simply observed without anesthesia. Previous studies have demonstrated that isoflurane anesthesia significantly influences brain FDG PET outcomes in small animals by decreasing glucose metabolism in the cortex and thalamus [31]. Consequently, the 8 h anesthesia might have led to an underestimation of the isolated effects of TH by reducing the uptake of ^18^F-FDG in certain areas of the rat brain. However, it is important to note that in clinical settings, the administration of sedative drugs is necessary during TH. The rationale behind this practice is based on the hypothesis that reducing the patient’s metabolic rate can improve the prognosis of cardiac arrest. Moreover, there is evidence to suggest that sedation contributes to the therapeutic effectiveness and outcomes of TH [32,33]. Although the absence of strict confounder control in this preclinical study could be viewed as a limitation, it also mirrors the complex clinical scenarios where separating TH from sedation is challenging.

Lastly, the results obtained from preclinical experiments may not directly translate to clinical environments. Additionally, the small sample size may not have been adequate for robust statistical analysis, potentially introducing bias. Particular caution should be exercised when interpreting the findings from the small subgroup of non-survivors due to the heightened risk of both false-positive and false-negative errors. Furthermore, before these findings can be considered for clinical application, several factors must be addressed. The availability of FDG PET is not uniform across all medical centers or clinical environments, and its cost may restrict access for certain populations or healthcare systems operating under financial constraints. Although in this study we did not perform CT-based attenuation correction for small-animal brain PET imaging, we believe this does not significantly compromise our results, considering factors such as the small size of rodents, tissue homogeneity, and the use of normalized SUV ratios. However, it is essential to note that attenuation correction is crucial for clinical applications.

## 5. Conclusions

This study provides novel insights into the effects of TH on brain glucose metabolism following CA using ^18^F-FDG PET imaging. Our findings reveal a nuanced impact of TH that varies with the severity of hypoxic ischemic encephalopathy. In cases of severe encephalopathy (the non-survived group), TH significantly improved neocortical relative glucose metabolism. However, this metabolic enhancement did not translate into improved survival outcomes, highlighting the limited efficacy of TH in severe cases. In mild encephalopathy cases (the survived group), TH showed no significant impact on brain glucose metabolism, functional outcomes, or survival rates. This suggests that TH may not provide additional benefits in less severe cases of post-CA brain injury.

## Figures and Tables

**Table 1:** Regional SUV according to survival.

	SUV		*p* Value
	Survived (*n* = 14)	Non-Survived (*n* = 7)	
Entorhinal Cortex	1.99 (1.45–2.70)	1.42 (1.11–1.56)	0.030 *
Frontal Association Cortex	2.23 (1.62–2.96)	1.31 (0.99–1.56)	0.002 *
Insular Cortex	2.37 (1.73–3.28)	1.59 (1.21–1.87)	0.007 *
Auditory Cortex	2.31 (1.76–3.20)	1.41 (1.14–1.87)	0.005 *
Cingulate Cortex	2.62 (1.92–3.54)	1.69 (1.20–1.97)	0.004 *
Medial Prefrontal Cortex	2.54 (1.91–3.42)	1.77 (1.30–1.94)	0.007 *
Motor Cortex	2.40 (1.74–3.29)	1.46 (1.08–1.81)	0.003 *
Orbitofrontal Cortex	2.56 (1.86–3.47)	1.65 (1.23–1.90)	0.004 *
Parietal Association Cortex	2.45 (1.85–3.34)	1.43 (1.11–1.87)	0.002 *
Retro-Splenial Cortex	2.26 (1.68–3.14)	1.43 (1.02–1.68)	0.003 *
Somatosensory Cortex	2.50 (1.79–3.43)	1.52 (1.15–1.91)	0.003 *
Visual Cortex	2.37 (1.81–3.30)	1.37 (1.07–1.82)	0.002 *
Olfactory Cortex	2.24 (1.61–3.05)	1.54 (1.17–1.72)	0.030 *

* *p*-values indicate significance at the 0.05 level.

**Table 2:** Regional SUVR_pons_ according to survival.

	SUVR_pons_		*p* Value
	Survived (*n* = 14)	Non-Survived (*n* = 7)	
Entorhinal Cortex	1.14 (1.12–1.17)	1.09 (1.07–1.13)	0.086
Frontal Association Cortex	1.25 (1.22–1.32)	1.01 (0.87–1.12)	0.001 *
Insular Cortex	1.38 (1.32–1.41)	1.23 (1.11–1.32)	0.001 *
Auditory Cortex	1.33 (1.31–1.37)	1.16 (1.05–1.25)	0.001 *
Cingulate Cortex	1.50 (1.46–1.55)	1.21 (1.13–1.45)	0.001 *
Medial Prefrontal Cortex	1.47 (1.42–1.52)	1.32 (1.18–1.44)	0.021 *
Motor Cortex	1.37 (1.33–1.42)	1.10 (0.99–1.28)	<0.001 *
Orbitofrontal Cortex	1.47 (1.42–1.50)	1.24 (1.13–1.38)	0.001 *
Parietal Association Cortex	1.40 (1.38–1.45)	1.12 (1.03–1.32)	0.001 *
Retro-Splenial Cortex	1.31 (1.25–1.37)	1.03 (0.96–1.23)	0.001 *
Somatosensory Cortex	1.41 (1.37–1.46)	1.17 (1.07–1.33)	<0.001 *
Visual Cortex	1.38 (1.34–1.40)	1.08 (1.00–1.27)	<0.001 *
Olfactory Cortex	1.28 (1.27–1.30)	1.18 (1.15–1.21)	0.014 *

* *p*-values indicate significance at the 0.05 level.

## Data Availability

Data will be made available on request.

## Supplementary Materials

The following supporting information can be downloaded at: https://www.mdpi.com/article/10.3390/diagnostics14151674/s1, Figure S1: Body temperature curves for 21 cardiac arrest rats; Figure S2: Overall survival in the TH and non-TH groups; Figure S3: Voxel-based comparison of SUV between survived and non-survived rats; Figure S4: Voxel-based comparison of SUVRpons between survived and non-survived rats; Figure S5: Voxel-based comparison of SUVRpons between TH and non-TH rats in the total group; Figure S6. Voxel-based comparison of SUVRpons between TH and non-TH rats in the survived subgroup; Table S1: MWM time and distance according to TH.

## Abbreviations

TH: therapeutic hypothermia; CA: cardiac arrest; OCHA: out-of-hospital cardiac arrest; CPR: cardiopulmonary resuscitation; ROSC: return of spontaneous circulation; VOI: volume of interest; SUV: standardized uptake value; FDR: false-discovery rate; MWM: Morris water maze.

## References

[1] Bernard S.A., Gray T.W., Buist M.D., Jones B.M., Silvester W., Gutteridge G., Smith K. (2002). Treatment of comatose survivors of out-of-hospital cardiac arrest with induced hypothermia. N. Engl. J. Med..

[2] Hypothermia after Cardiac Arrest Study Group (2002). Mild therapeutic hypothermia to improve the neurologic outcome after cardiac arrest. N. Engl. J. Med..

[3] Field J.M., Hazinski M.F., Sayre M.R., Chameides L., Schexnayder S.M., Hemphill R., Samson R.A., Kattwinkel J., Berg R.A., Bhanji F. (2010). Part 1: Executive Summary: 2010 American Heart Association guidelines for cardiopulmonary resuscitation and emergency cardiovascular care. Circulation.

[4] Kim Y.-M., Jeung K.W., Kim W.Y., Park Y.S., Oh J.S., You Y.H., Lee D.H., Chae M.K., Jeong Y.J., Kim M.C. (2021). 2020 Korean Guidelines for Cardiopulmonary Resuscitation. Part 5. Post-cardiac arrest care. Clin. Exp. Emerg. Med..

[5] Sekhon M.S., Ainslie P.N., Griesdale D.E. (2017). Clinical pathophysiology of hypoxic ischemic brain injury after cardiac arrest: A “two-hit” model. Crit. Care.

[6] McCullough J.N., Zhang N., Reich D.L., Juvonen T.S., Klein J.J., Spielvogel D., Ergin M., Griepp R.B. (1999). Cerebral metabolic suppression during hypothermic circulatory arrest in humans. Ann. Thorac. Surg..

[7] Webster C.M., Kelly S., Koike M.A., Chock V.Y., Giffard R.G., Yenari M.A. (2009). Inflammation and NFκB activation is decreased by hypothermia following global cerebral ischemia. Neurobiol. Dis..

[8] Panchal A.R., Bartos J.A., Cabañas J.G., Donnino M.W., Drennan I.R., Hirsch K.G., Kudenchuk P.J., Kurz M.C., Lavonas E.J., Morley P.T. (2020). Part 3: Adult basic and advanced life support: 2020 American Heart Association Guidelines for cardiopulmonary resuscitation and emergency cardiovascular care. Circulation.

[9] Nielsen N., Friberg H., Gluud C., Herlitz J., Wetterslev J. (2011). Hypothermia after cardiac arrest should be further evaluated—A systematic review of randomised trials with meta-analysis and trial sequential analysis. Int. J. Cardiol..

[10] Garrido C.C., Gallego B.R., García J.C.S., Martín J.C., Troya M.M., Blanque R.R. (2021). The effect of therapeutic hypothermia after cardiac arrest on the neurological outcome and survival—A systematic review of RCTs published between 2016 and 2020. Int. J. Environ. Res. Public Health.

[11] Dankiewicz J., Cronberg T., Lilja G., Jakobsen J.C., Levin H., Ullén S., Rylander C., Wise M.P., Oddo M., Cariou A. (2021). Hypothermia versus normothermia after out-of-hospital cardiac arrest. N. Engl. J. Med..

[12] Fink E.L., Clark R.S., Berger R.P., Fabio A., Angus D.C., Watson R.S., Gianakas J.J., Panigrahy A., Callaway C.W., Bell M.J. (2018). 24 vs. 72 hours of hypothermia for pediatric cardiac arrest: A pilot, randomized controlled trial. Resuscitation.

[13] Chevin M. (2021). Therapeutic Hypothermia and Interleukin-1 Blockade as Neuroprotective Strategies in Neonatal Encephalopathy and Arterial Ischemic Stroke. Ph.D. Thesis.

[14] Nakamura T., Kuroda Y., Torigoe N., Abe Y., Yamashita S., Kawakita K., Kawai N., Tamiya T., Itano T., Nagao S. (2009). Cerebral metabolism monitoring during hypothermia following resuscitation from cardiopulmonary arrest. Acta Neurochir. Suppl..

[15] Kim D., Yoon H.-J., Lee W.J., Woo S.H., Kim B.S. (2019). Prognostic value of 18F-FDG brain PET as an early indicator of neurological outcomes in a rat model of post-cardiac arrest syndrome. Sci. Rep..

[16] Kim D., Lee W.J., Lee H.W., Kim B.S., Woo S.H., Yoon H.-J. (2022). Application of ^18^F-FDG brain PET for survival prediction in a rat model of hanging-induced hypoxic brain injury. Ann. Nucl. Med..

[17] Kilkenny C., Browne W., Cuthill I.C., Emerson M., Altman D.G., NC3Rs Reporting Guidelines Working Group (2010). Animal research: Reporting in vivo experiments: The ARRIVE guidelines. Br. J. Pharmacol..

[18] Yu S., Wu C., Zhu Y., Diao M., Hu W. (2023). Rat model of asphyxia-induced cardiac arrest and resuscitation. Front. Neurosci..

[19] Wang X., Li M., Yang Z., Li H., Wang Y., Tang W., Wu Y., Xiao P., Jiang S., Shi Q. (2021). Comparison of the Protective Effect of Different Mild Therapeutic Hypothermia Temperatures on Intestinal Injury After Cardiopulmonary Resuscitation in Rats. Shock.

[20] Simpson D.P. (1997). Prolonged (12 hours) intravenous anesthesia in the rat. Lab. Anim. Sci..

[21] Vorhees C.V., Williams M.T. (2006). Morris water maze: Procedures for assessing spatial and related forms of learning and memory. Nat. Protoc..

[22] Putzu A., Valtorta S., Di Grigoli G., Haenggi M., Belloli S., Malgaroli A., Gemma M., Landoni G., Beretta L., Moresco R.M. (2018). Regional differences in cerebral glucose metabolism after cardiac arrest and resuscitation in rats using [18F] FDG positron emission tomography and autoradiography. Neurocrit. Care.

[23] Schaafsma A., de Jong B., Bams J., Haaxma-Reiche H., Pruim J., Zijlstra J. (2003). Cerebral perfusion and metabolism in resuscitated patients with severe post-hypoxic encephalopathy. J. Neurol. Sci..

[24] Rudolf J., Ghaemi M., Ghaemi M., Haupt W.F., Szelies B., Heiss W.-D. (1999). Cerebral glucose metabolism in acute and persistent vegetative state. J. Neurosurg. Anesthesiol..

[25] Li Y.-Q., Liao X.-X., Lu J.-H., Liu R., Hu C.-L., Dai G., Zhang X.-S., Shi X.-C., Li X. (2015). Assessing the early changes of cerebral glucose metabolism via dynamic 18FDG-PET/CT during cardiac arrest. Metab. Brain Dis..

[26] Zhang H.J., Mitchell S., Fang Y.-H., Tsai H.-M., Piao L., Ousta A., Leoni L., Chen C.-T., Sharp W.W. (2021). Assessment of Brain Glucose Metabolism Following Cardiac Arrest by [^18^F]FDG Positron Emission Tomography. Neurocrit. Care.

[27] Tintinalli J.E. (1992). Emergency Medicine: A Comprehensive Study Guide.

[28] Toyama H., Ichise M., Liow J.-S., Modell K.J., Vines D.C., Esaki T., Cook M., Seidel J., Sokoloff L., Green M.V. (2004). Absolute quantification of regional cerebral glucose utilization in mice by 18F-FDG small animal PET scanning and 2-14C-DG autoradiography. J. Nucl. Med..

[29] Thorngren-Jerneck K., Ohlsson T., Sandell A., Erlandsson K., Strand S.-E., Ryding E., Svenningsen N.W. (2001). Cerebral glucose metabolism measured by positron emission tomography in term newborn infants with hypoxic ischemic encephalopathy. Pediatr. Res..

[30] Edison P., Raza S., Femminella G.D., Blunt E.G., Carver S., Livingston N.R., Nowell J., Holmes C., Ritchie C.W., Lawrence R.M. (2020). Relationship between spectral analysis, SUV and SUV Pons ratio as a measure of cerebral glucose metabolic rate in Alzheimer’s disease: Neuroimaging/multi-modal Comparisons. Alzheimer’s Dement..

[31] Park T.Y., Nishida K.S., Wilson C.M., Jaiswal S., Scott J., Hoy A.R., Selwyn R.G., Dardzinski B.J., Choi K.H. (2017). Effects of isoflurane anesthesia and intravenous morphine self-administration on regional glucose metabolism ([^18^F]FDG-PET) of male Sprague-Dawley rats. Eur. J. Neurosci..

[32] Samaniego E.A., Mlynash M., Caulfield A.F., Eyngorn I., Wijman C.A.C. (2011). Sedation confounds outcome prediction in cardiac arrest survivors treated with hypothermia. Neurocrit. Care.

[33] Dell’Anna A.M., Taccone F.S., Halenarova K., Citerio G. (2014). Sedation after cardiac arrest and during therapeutic hypothermia. Minerva Anestesiol..

