# Supplementary material for: The Effect of Therapeutic Hypothermia on Ischemic Brain Injury in a Rat Model of Cardiac Arrest: An Assessment Using 18F-FDG PET
Source: Diagnostics (Basel). 2024 Aug 2;14(15):1674. doi: 10.3390/diagnostics14151674 (PMC11311465; doi:10.3390/diagnostics14151674)
Supplement: Supplementary file 1 [file diagnostics-14-01674-s001.zip › Supplemental Figure.pdf]

Supplemental Figure S1. Body temperature curves for 21 cardiac arrest rats

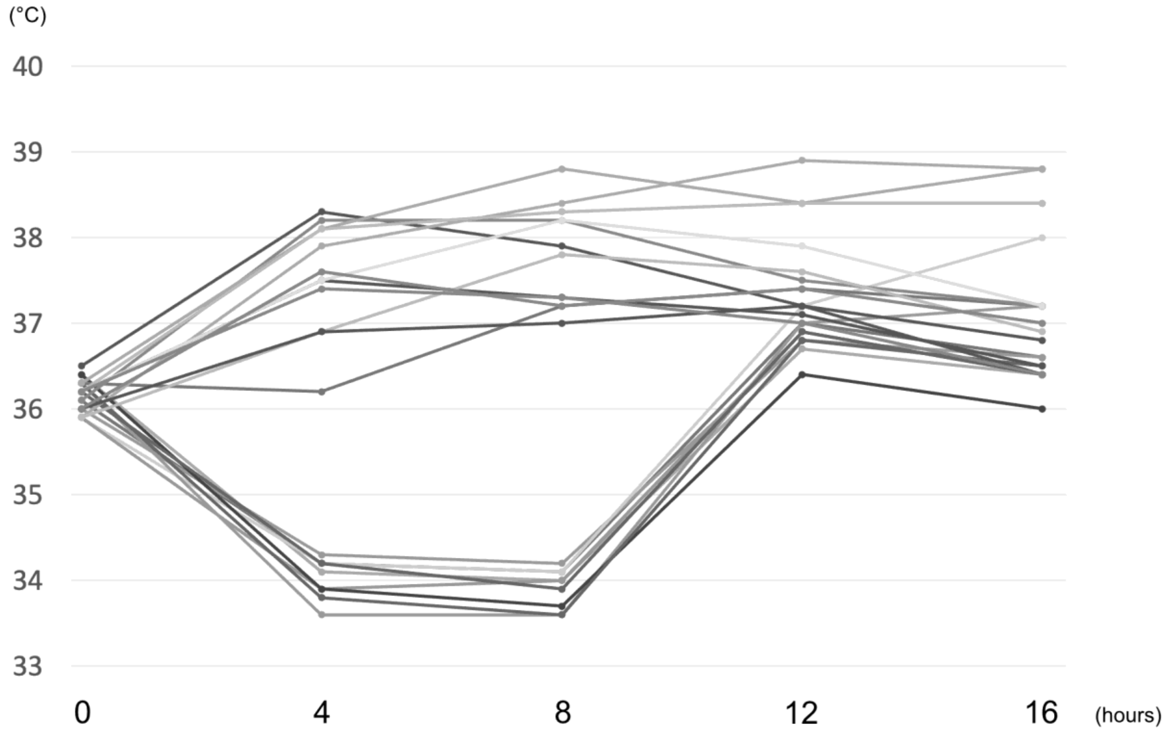

Supplemental Figure S2. Overall survival in the TH and non-TH groups

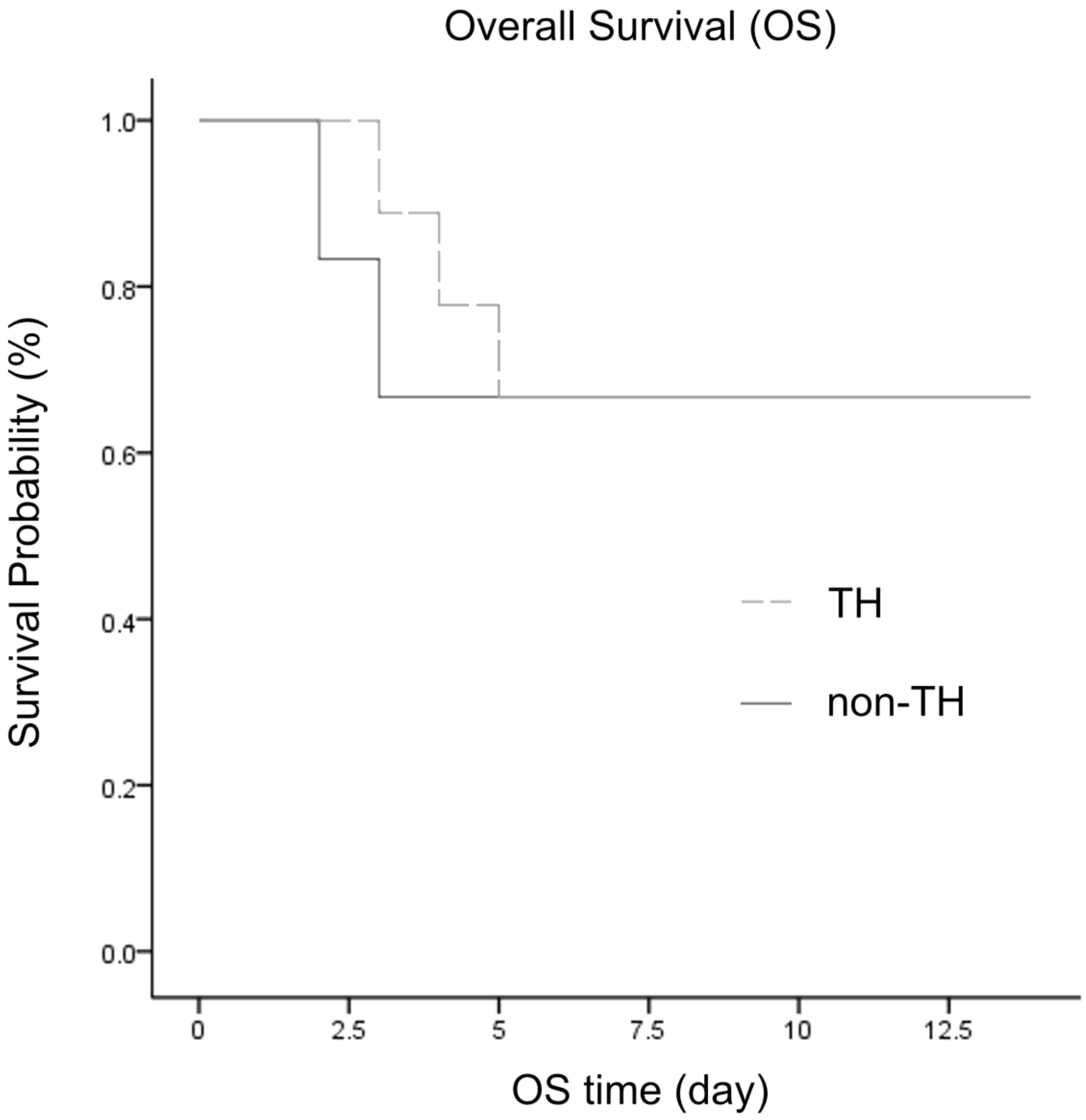

Supplemental Figure S3. Voxel-based comparison of SUV between survived and non-survived rats

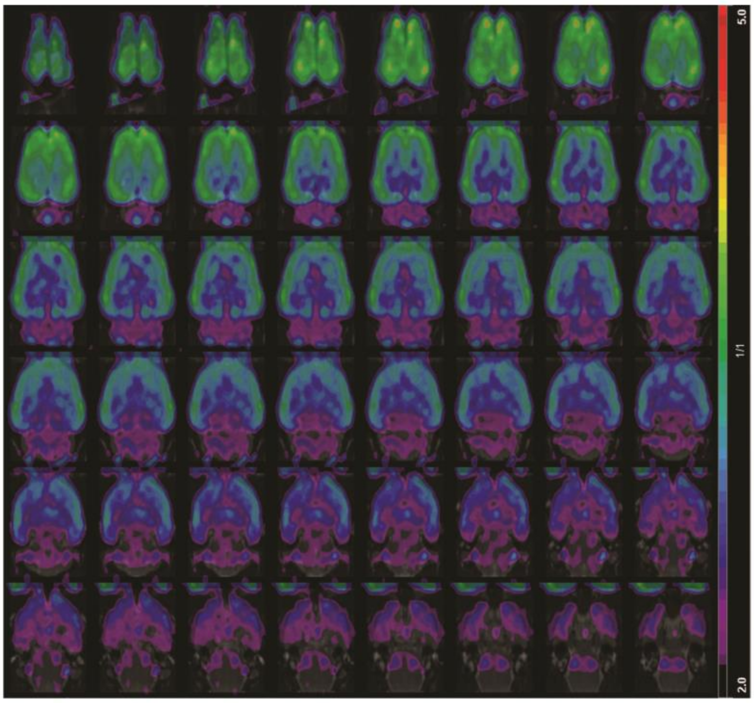

The statistical parametric map of “non-survived < survived” obtained from 18F-FDG PET showed a global decrease in SUV in non-survived rats (FDR-corrected $p < 0.05$, $t > 2.2978$).

Supplemental Figure S4. Voxel-based comparison of SUVRpons between survived and non-survived rats

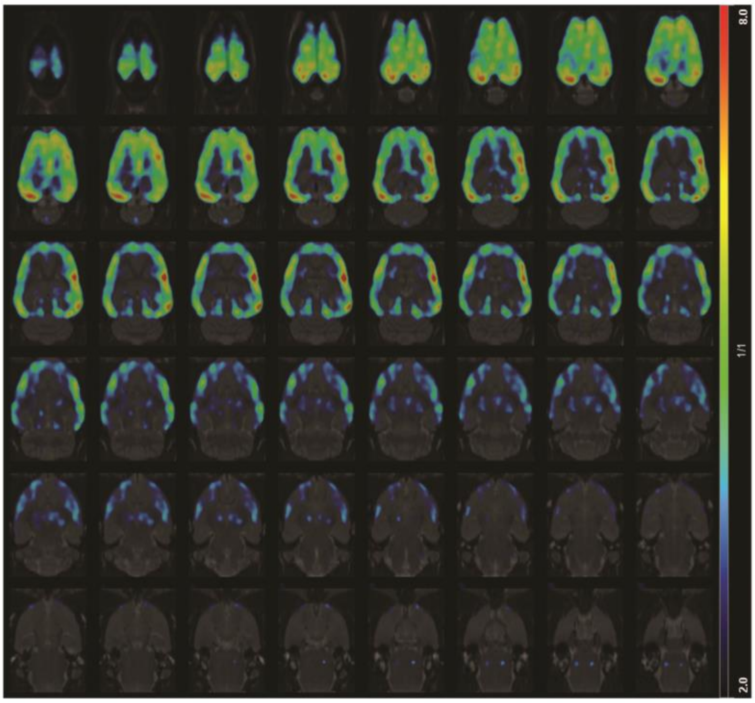

The statistical parametric map of “non-survived < survived” obtained from 18F-FDG PET normalized to the pons showed a global decrease in SUVRpons in non-survived rats (FDR-corrected $p < 0.05$, $t > 2.2943$).

Supplemental Figure S5. Voxel-based comparison of SUVRpons between TH and non-TH rats in the total group

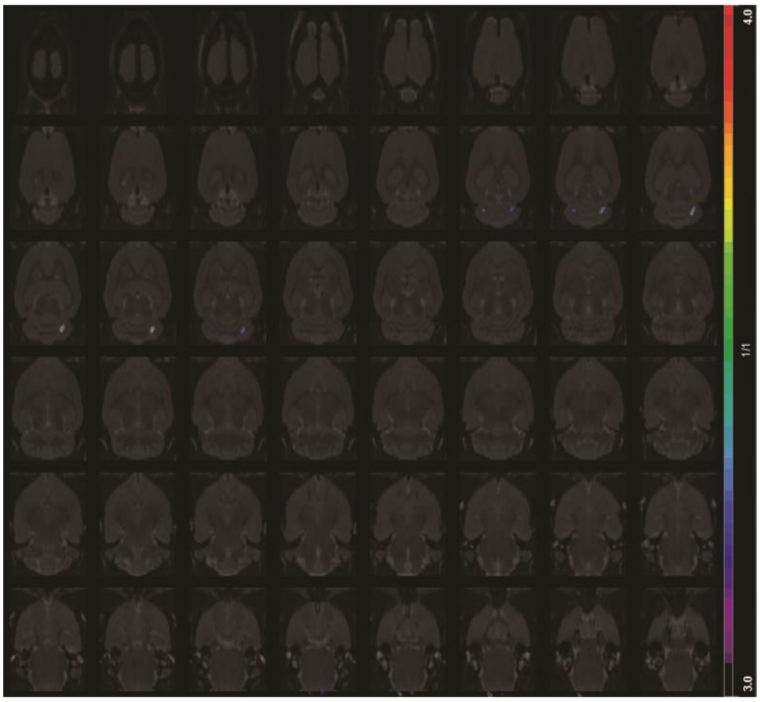

The statistical parametric map of “non-TH < TH” obtained from 18F-FDG PET normalized to the pons showed no significant difference in the cortical SUVRpons according to TH application in the total group (uncorrected $p < 0.001$, $t > 3.5794$).

Supplemental Figure S6. Voxel-based comparison of SUVRpons between TH and non-TH rats in the survived subgroup

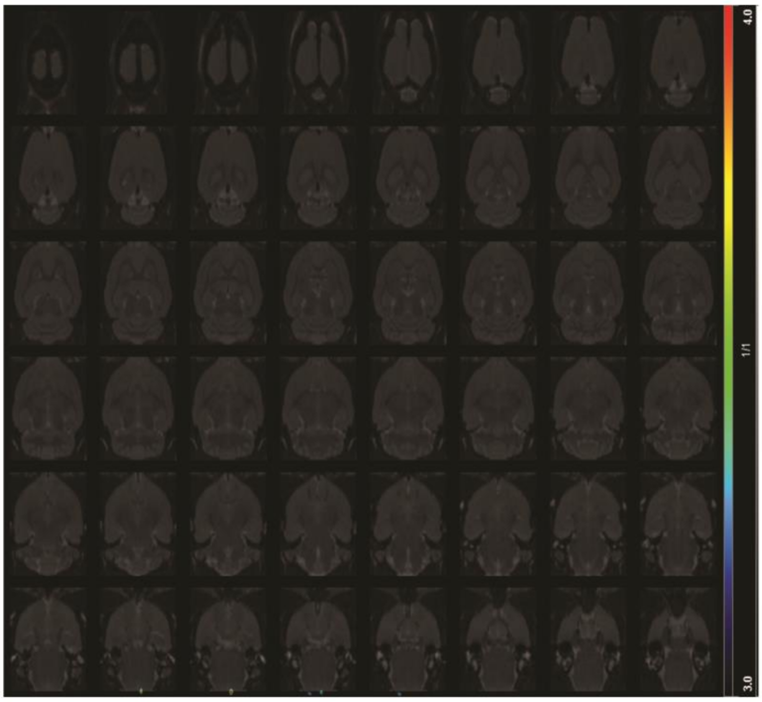

The statistical parametric map of “non-TH < TH” obtained from 18F-FDG PET normalized to the pons showed no significant difference in the cortical SUVRpons according to TH application in the survived subgroup (uncorrected $p < 0.001$, $t > 3.9296$).
