# Supplementary material for: The Effect of Therapeutic Hypothermia on Ischemic Brain Injury in a Rat Model of Cardiac Arrest: An Assessment Using 18F-FDG PET
Source: Diagnostics (Basel). 2024 Aug 2;14(15):1674. doi: 10.3390/diagnostics14151674 (PMC11311465; doi:10.3390/diagnostics14151674)
Supplement: Supplementary file 1 [file diagnostics-14-01674-s001.zip › Supplemental Table 1.pdf]

Supplemental Table S1. MWM time and distance according to the TH

Subject	Before Intervention		TH		Non-TH	
	Time (Seconds)	Distance (cm)	Time (Seconds)	Distance (cm)	Time (Seconds)	Distance (cm)
No 1.	8.7	125.8	9.7	145.2		
No 2.	9.3	172.3	9.9	197.7		
No 3.	8.1	111.9	8.5	119.2		
No 4.	8.6	135.7	9.3	173.5		
No 5.	9.9	189.1	11.5	250.4		
No 6.	9.3	166.5	10.2	217.2		
No 7.	9.2	141.7			10.5	225.2
No 8.	9.4	163.9			10.3	215.7
No 9.	9.1	171.4			9.3	157.3
No 10.	9.7	159.2			11.4	246.8
No 11.	9.2	109.5			9.3	140.7
No 12.	9.6	115.6			10.5	153.2
No 13.	9.3	179.2			9.6	175.9
No 14.	9.8	185.7			11.3	245.3

MWM, Morris water maze; TH, therapeutic hypothermia
