# Supplementary figures and images for: The Effect of Therapeutic Hypothermia on Ischemic Brain Injury in a Rat Model of Cardiac Arrest: An Assessment Using 18F-FDG PET
Source: Diagnostics (Basel). 2024 Aug 2;14(15):1674. doi: 10.3390/diagnostics14151674 (PMC11311465; doi:10.3390/diagnostics14151674)

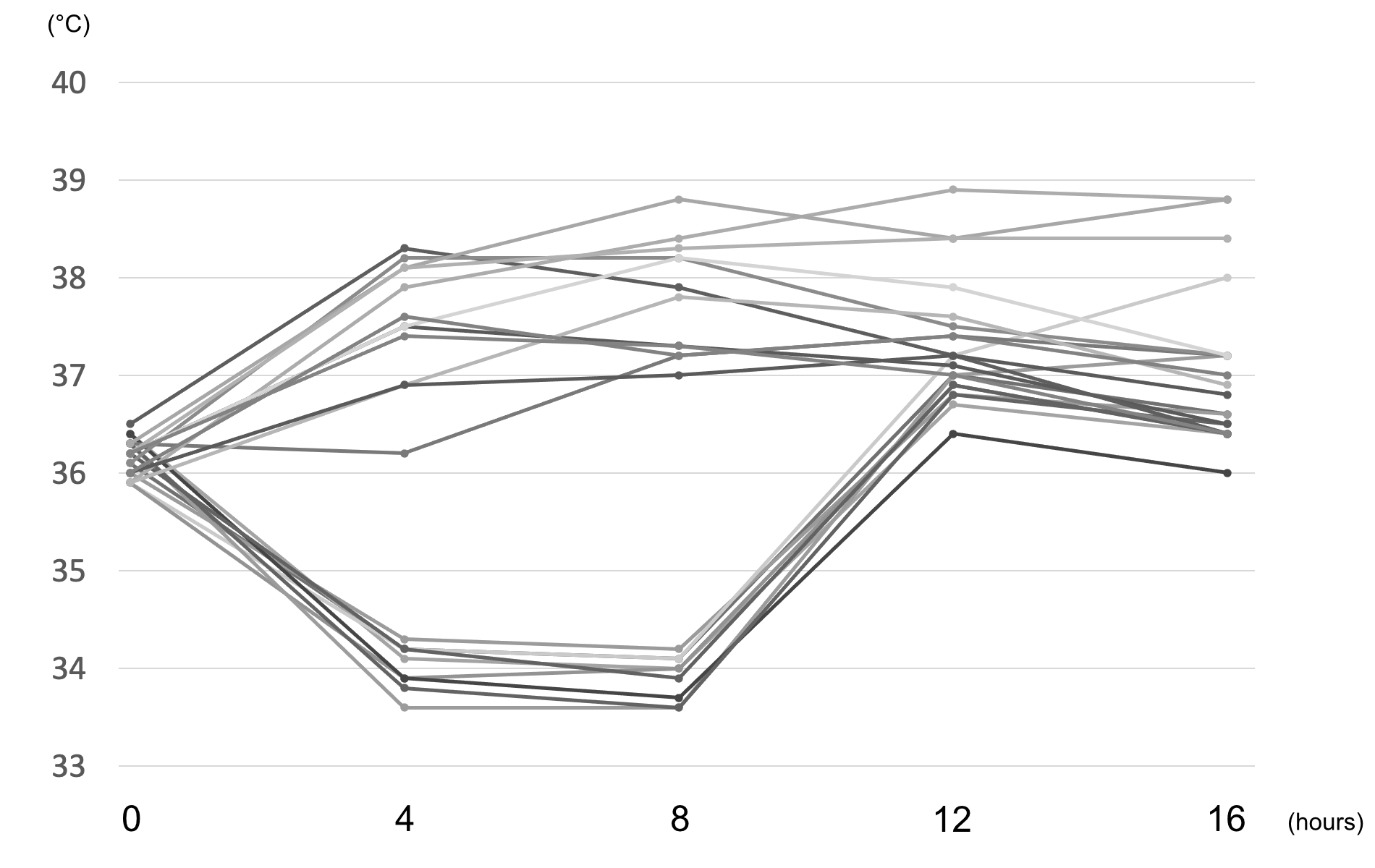

Supplement: Supplementary file 1 [file diagnostics-14-01674-s001.zip › S_Fig1.tiff]

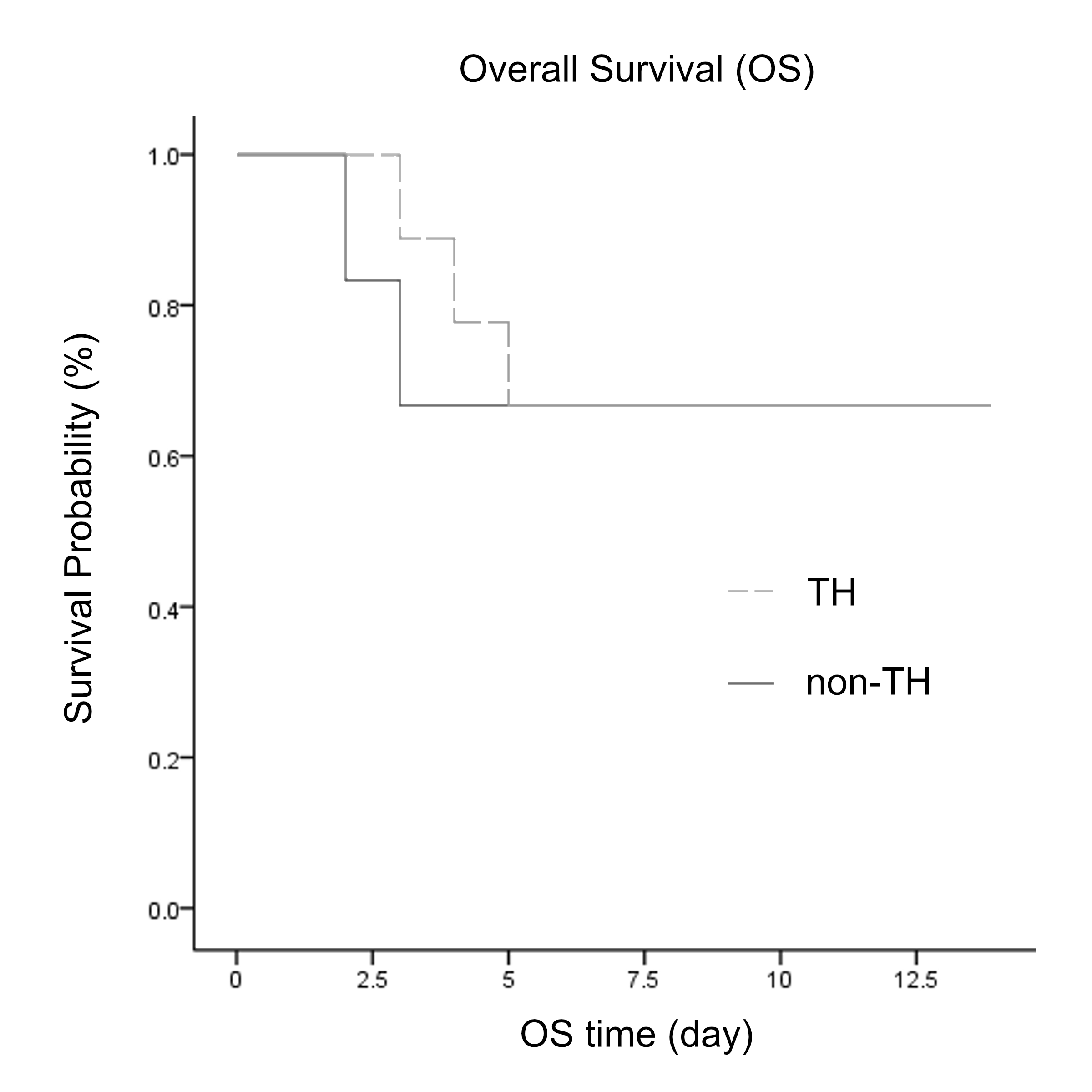

Supplement: Supplementary file 1 [file diagnostics-14-01674-s001.zip › S_Fig2.tiff]

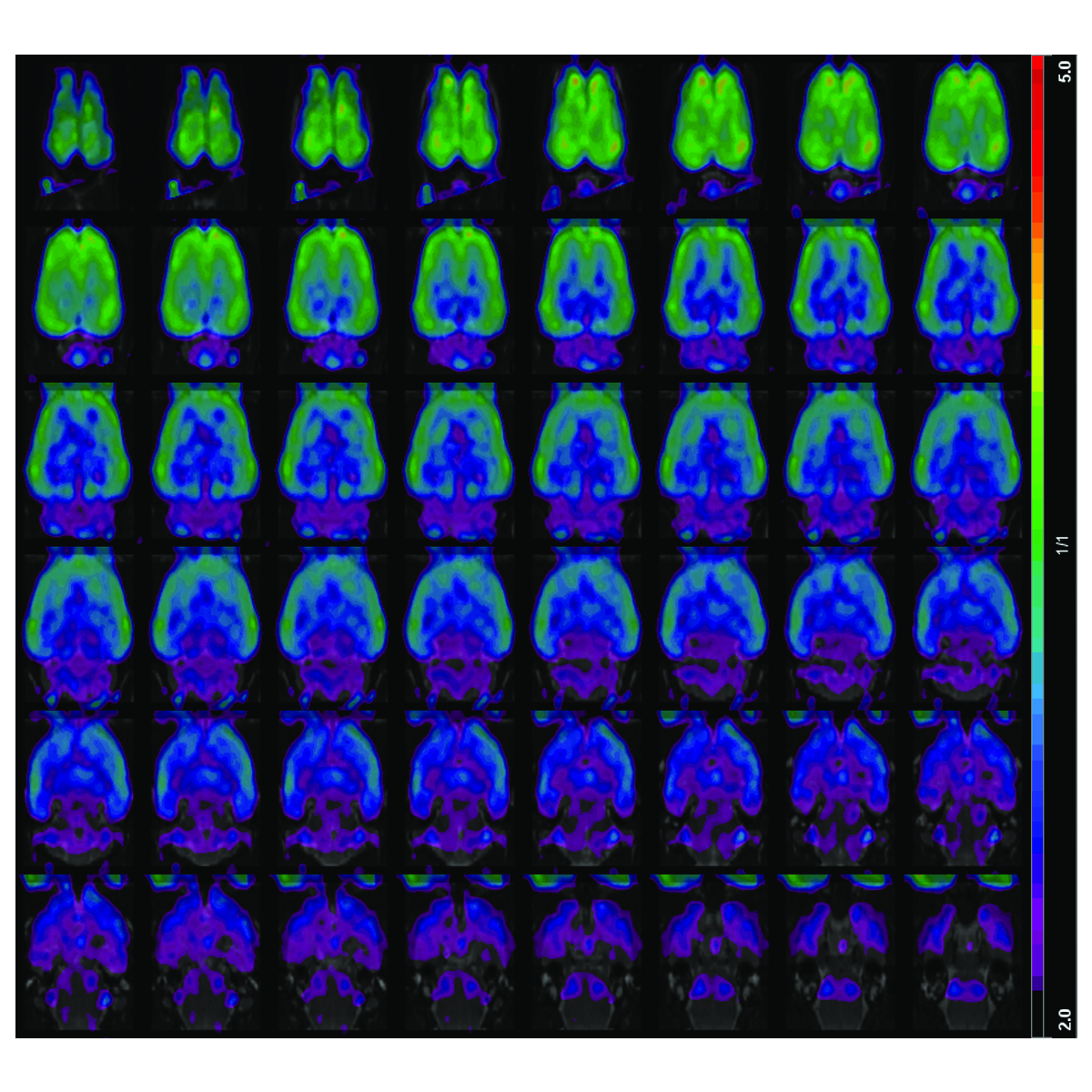

Supplement: Supplementary file 1 [file diagnostics-14-01674-s001.zip › S_Fig3.tiff]

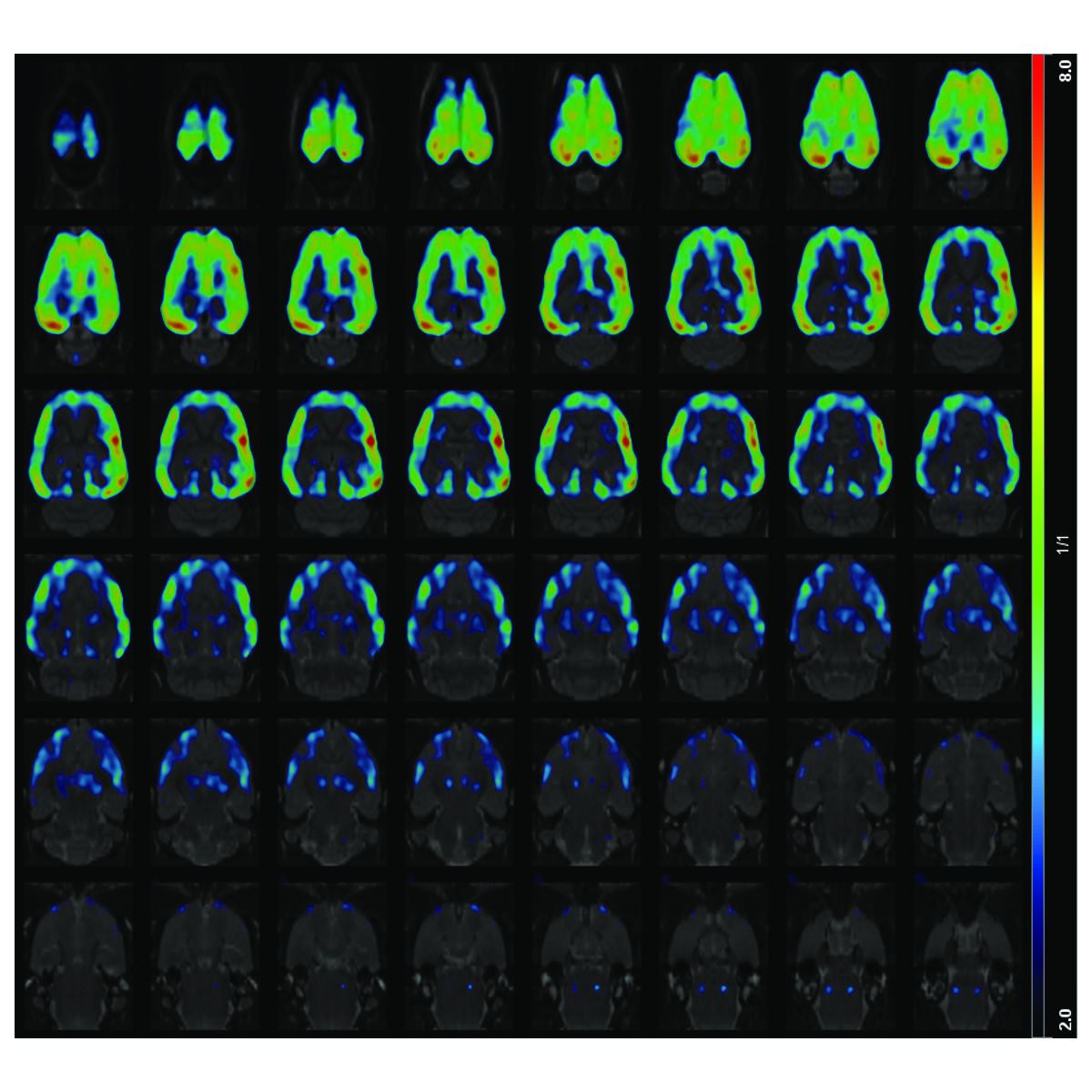

Supplement: Supplementary file 1 [file diagnostics-14-01674-s001.zip › S_Fig4.tiff]

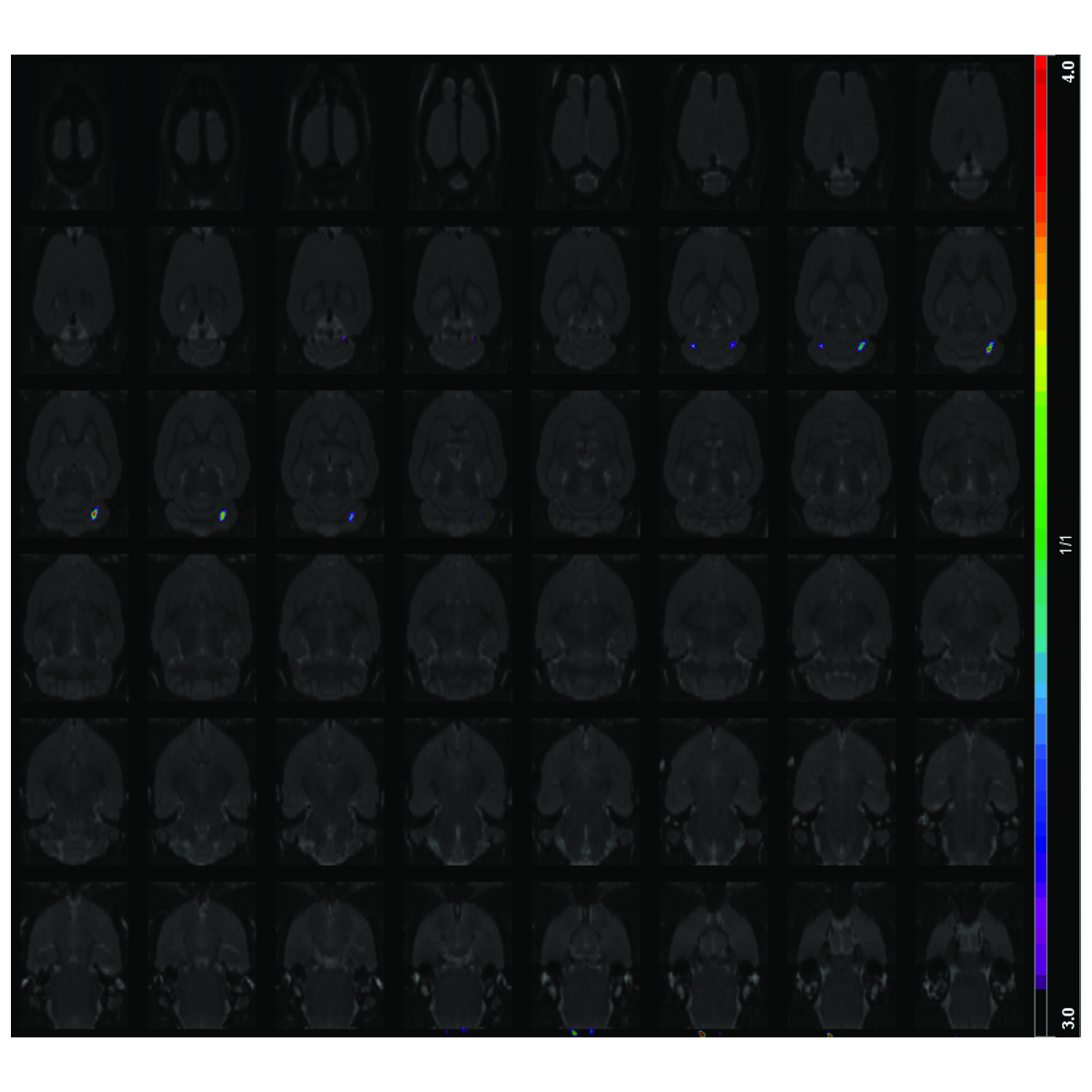

Supplement: Supplementary file 1 [file diagnostics-14-01674-s001.zip › S_Fig5.tiff]

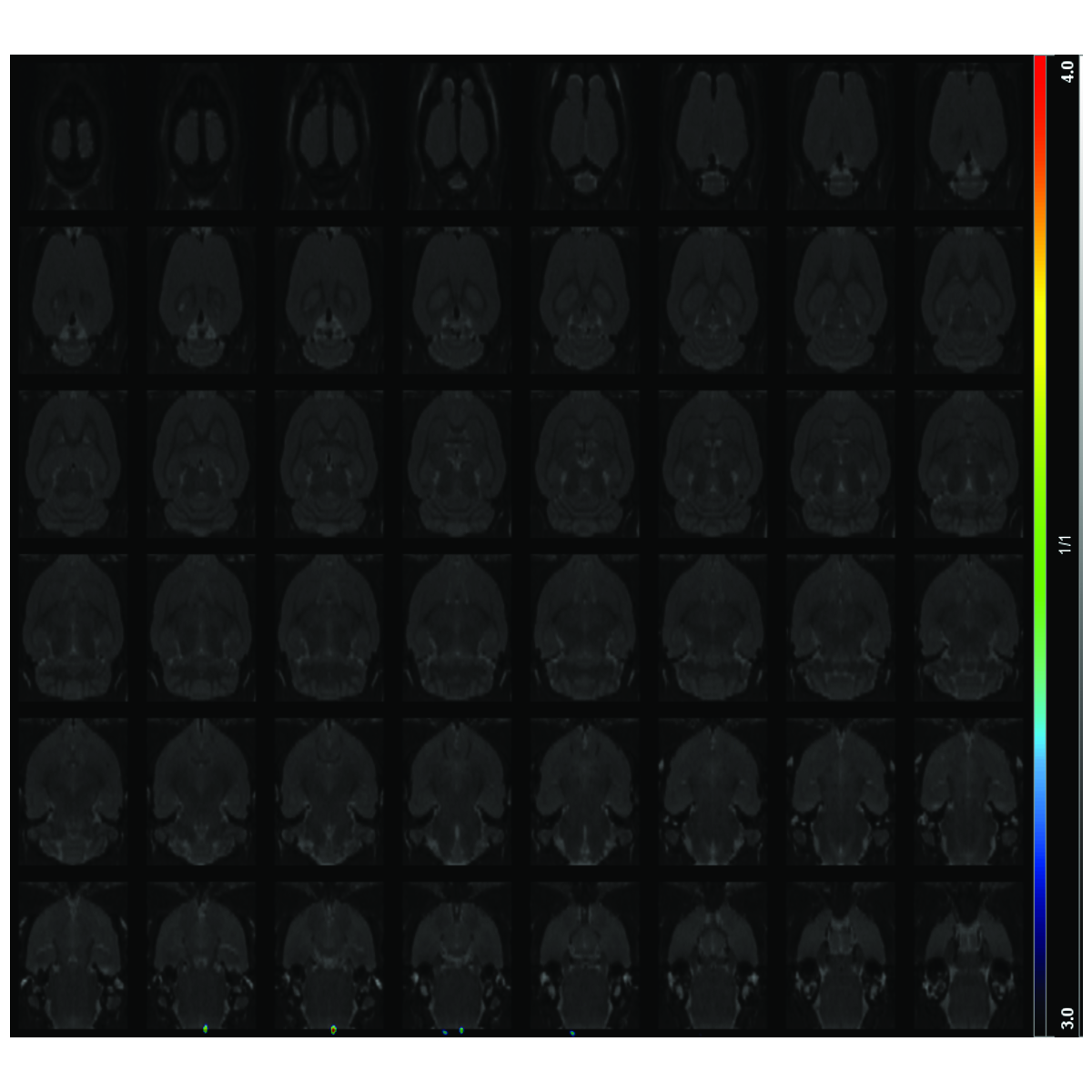

Supplement: Supplementary file 1 [file diagnostics-14-01674-s001.zip › S_Fig6.tiff]
